# COVID-19 and Mental Health: A Study of Stress, Resilience, and Depression among the Older Population in Pakistan

**DOI:** 10.3390/healthcare9040424

**Published:** 2021-04-06

**Authors:** Ayesha Mumtaz, Faiza Manzoor, Shaoping Jiang, Mohammad Anisur Rahaman

**Affiliations:** 1 College of Public Administration, Zhejiang University, Hangzhou 310058, China; ayeshamumtaz04@gmail.com (A.M.); anisrahaman01@gmail.com (M.A.R.); 2 Department of Agricultural Economics and Management, School of Public Affairs, Zhejiang University, Hangzhou 310058, China; 3 Guanghua Law School, Zhejiang University, Zhijiang Road, Xihu District, Hangzhou 310008, China; 4 Department of Sociology, Bangabandhu Sheikh Mujibur Rahman Science and Technology University, Gopalganj 8100, Bangladesh

**Keywords:** fear of COVID-19, exposure to COVID-19, self-efficacy, depression, elderly people

## Abstract

Extending studies of the adverse effects of SARS-2 coronavirus on general health consequences, this research explores complexities related to the mental health of the elderly as a result of pandemic-related stress. The study addresses this issue by using resilience theory to examine the effects of fear and exposure related to COVID-19 and depression. Besides, our study examines the moderating effects of self-efficacy in order to provide an understanding of how the coping abilities of the elderly may mitigate the effect of stress levels on mental health during pandemics. Our model is tested by analysing the survey data collected from Rawalpindi, a metropolitan city in Pakistan. The main results of the study confirm the positive association of pandemic-related fear and exposure virus with depression. However, self-efficacy shows a negative direct effect on depression, and the findings also confirm the moderation effect of self-efficacy on the fear of COVID-19 and depression, but the moderation effect of self-efficacy on COVID-19 exposure and depression is not supported. Based on the outcomes, some severe geriatric care policies that could weaken the pandemic-related fear, exposure to the virus, and depression are recommended.

## 1. Introduction

Mental health problems are of major global public health concerns during pandemics. The SARS-2 coronavirus originated from Southeast Asia and spread worldwide, having adverse physical and mental health impacts on all age groups, resulting in over 132 million infected and over 2.8 million deaths worldwide to date [1]. Even though the virus transmission rate is reported to be similar for all age groups, devastating evidence from around the world suggests that its mortality rates and progression risk are significantly higher in older people [2]. In light of early data from China, the case fatality rate in elderly patients varies between 1.3% and 14.8% [3]. Furthermore, it is well known that social isolation among older adults is a “serious public health concern” because of the risk of cardiovascular, autoimmune, neurocognitive, and mental health problems [4]. The integral role of mental health on general wellness has also been acknowledged by the World Health Organization (WHO). Increasing mental health problems have led to higher rates of disability, declines in quality of life, and increased mortality ratios among elderly people in the past few years. Thus, addressing this issue has become a worldwide challenge worldwide [5,6]

To further manage mental health issues during pandemics, the topic of the fear of COVID-19 and deteriorating psychological health has been receiving attention in European and few East Asian countries [7,8,9,10,11], with researchers largely focusing on the situation of fear and anxiety associated with COVID-19 and mental health in a variety of contexts. However, studies on psychological distress caused by the COVID-19 pandemic in the older population are scarce. In addition, the evidence is also not uniformly distributed, with more COVID-19 and mental health-related studies conducted in a few countries from East Asia and Europe [11,12,13,14], in comparison to South Asia where this topic has received little or no attention to date. Part of the reason for this skewed distribution of evidence concerning pandemic-related depression in the elderly population is the varying availability of data on this subject.

One conclusion based on the existing literature about COVID-19-related fear and exposure and depression is that the experiences of elderly people in various settings are heterogeneous. Some studies show adverse effects on mental health due to fear and exposure to COVID-19 [7,10,14], while other reports no psychiatric morbidity due to COVID-19 [15].

Following the outbreak, the Pakistani government has tightened border controls and implemented social distancing measures; as numbers of cases locally and worldwide increased, the Pakistani government implemented a partial lockdown in early March 2020 across the country [16]. Jobs were affected, with many workers left without a job or furloughed as the economy ground to a halt. With prolonged quarantines, restrictions on public life, and worries surrounding job security, there follows the risk of rising levels of psychiatric issues such as anxiety, stress, and depression amongst the population.

Currently, to our knowledge, there are no studies on the psychological impact of the novel coronavirus disease on the older population in Pakistan. This paper aims to address pandemic-related fear and depression amongst the older population, as well as to explore the relationship between the self-efficacy of elderly people and their depression levels during COVID-19. Therefore, additional studies that explore the specific situation in a particular country are necessary.

The main objective of this study was to highlight the effects of the stressors related to COVID-19 on the mental health of older people. Whether COVID-19 fear and exposure have a positive or negative impact on the mental health of the elderly depends on some external factors that we explore in the context of Pakistan. As with analyses of this kind, endogeneity is an issue, regarding whether those with high-level coping abilities are less likely to suffer from depression. It is widely acknowledged that cognitive abilities relevant to self-control play a major role in both psychological adjustment and physical health [17,18]. Constructs relating to self-control expectancies, such as self-efficacy [17], occupy prominent positions in contemporary theoretical formulations of coping and behavioral efficacy. It has been demonstrated that self-efficacy is an important factor with regards to an individual’s perceptions of his or her ability to perform specific health behaviors, which greatly influence actual health behaviors and status [17,19]. Individuals with high coping traits, e.g., self-efficacy, are more likely to seek preventive care and rate their health more favorably than individuals with low self-efficacy [17,20]. Therefore, in this study, we used self-efficacy as a moderator to show the effect of the resilient factor as a moderator. The findings from this study are the first in-depth study in Pakistan. The specific research questions of the study are:

1. Do COVID-19-related stressors, such as fear of and exposure to COVID-19, affect the mental health of elderly people?
2. Do coping abilities like self-efficacy play a moderating role between fear of COVID-19, exposure to COVID-19 and depression?

In the following, we provide brief theoretical background and review of the literature, present methods and measures of the study, and elaborate the survey findings in detail. In the last section, a discussion of the results, limitations and implications of the research concludes our study.

## 2. Theoretical Background

Self-efficacy, as a resilient cognitive variable, has been used extensively within the framework of resilience theory. In particular, resilience theory is one of the widely used theoretical paradigms in the field of psychology, where researchers have explained several mechanisms by which environmental and individual factors help to reduce or offset the adversity of stressors or risk factors [21]. Resilience theory relates to “the process of adapting well in the face of adversity, trauma, tragedy, threats, or even significant sources of stress” [22]. With regard to COVID-19 pandemic, the protective factor model of resilience theory is considered appropriate as it defines cognitive traits as protective factors or resilience factors as moderators between the risk and consequences [23]. In general terms, elderly people are more vulnerable to depression in response to adverse situations [24]. However, the situation may differ for those who have strong cognitive abilities, as the previous literature has supported [25], that individual’s capability to believe in their own abilities reduces their depression level. In addition, the studies also used self-efficacy as the cognitive trait to support the positive consequences in responding to adverse situations [26,27]. Hence, based on the above arguments, it is assumed that elderly people who have strongly perceived self-efficacy are more likely to show fewer depressive symptoms in response to pandemic situations.

### 2.1. Fear and Exposure of COVID-19

The emergence of the SARS-2 coronavirus pandemic at the end of 2019 attracted researchers to conduct studies on the psychological distress caused by the fear of COVID-19. Anxiety and fear related to pandemics is a natural response reported by several studies to date [7,8,28]. Fear is reported as one of the characteristics of infectious diseases, which along with comorbidities further leads to psychological challenges during pandemics [28]. The concept of the fear of COVID-19 has attracted attention since the virus originated. Initially, Daniel Ahorsu, along with his colleagues, developed a fear of the COVID-19 scale, particularly to assess the psychometric nature of this concept based on the existing scales of fear and experts’ evaluations [28]. Later on, this scale was implemented in diverse settings to investigate the psychological effects of fear of COVID-19 [29]. Fear of COVID-19 may lead to adverse consequences, which can negatively affect psychological health and life satisfaction [29]. Researchers have also reported that the fear of COVID-19 may cause irrational thoughts leading to depression [28].

The effect of the level of fear and anxiety related to COVID-19 varies among different cultures and age-groups. Studies conducted in Brazil [30] and Germany [7] reported a higher level of COVID-19 fear among women and younger individuals as compared to males and the elderly group. Whereas a study conducted in Spain reported more depression and anxiety symptoms in people more than 64 years old [5]. In terms of the cultural background, a survey study conducted in Eastern Europe [31] indicated less fear related to the COVID-19 as compared to an Iranian sample [32]. In contrast, the existing mental health literature in China during the COVID-19 outbreak, where similar psychological measurement tools were used, could not observe any difference between genders, but younger individuals reported more anxiety symptoms.

Higher exposure to COVID-19 is also considered a risk factor that may cause depression among people during the pandemic. Studies found that being worried about infection caused by coronavirus [10] and having more contact with relatives is associated with declined mental health during the quarantine period in pandemic [7]. Similarly, a study conducted in Spain reported high depression in the presence of COVID-19 symptoms and having close contact with a confirmed patient of COVID-19 [14].

### 2.2. COVID-19 and Depression

Research studies have mainly reported adverse mental health impacts due to stress factors associated with COVID-19 [10,14,33]. A variety of factors associated with the high risk of mental issues during the COVID-19 pandemic have been reported by several researchers. In May 2020, a review study aimed to explore the psychological symptoms associated with the pandemic among the general population and healthcare workers [34]. The authors reviewed 43 research studies to investigate the association between COVID-19 and mental health. Similar findings have been reported by another review study with a higher prevalence of depression (28%) and anxiety (33%) among health care workers and the general population [35]. In Asia, the majority of the studies have been conducted on the Chinese population. In China, a study conducted at the start of the outbreak reported that 58.3% of Chinese participants were depressed. A similar study revealed that some factors, like timely and up to date information regarding the COVID-19, were associated with less stress, anxiety, and depression [11]. Studies conducted in Europe also reported several COVID-19 risk factors associated with depression. Such as, a German study [7], conducted on the general population during COVID-19, revealed higher levels of depression (14.7%), psychological distress (65.2%), and general anxiety (44.9%).

Therefore, it seems that there are risk factors for depression irrespective of gender, age, nationality, or culture. Most of the studies, however, focus on the general audience rather than the specific age group. Elderly people with several risks factors, such as old age stigmatization, having chronic morbidities, less social support, living alone, and a higher level of depression, are more likely to suffer from COVID-19 [4]. Hence, to protect the elderly from the adverse effects of the pandemic, it is important to explore the potential mental health problems that may occur within this group and adopt the required measures.

### 2.3. COVID-19 and Self-Efficacy

Self-efficacy is defined as an “individuals’ beliefs in their capabilities to exercise control over challenging demands and over their own functioning” [36]. Self-efficacy has been considered as a regulatory function in various health sectors, inducing increased compliance with medical recommendations (for example, adopting an active lifestyle physically), coping with positive and negative influences, and the better management of pain or stress. Bandura in 1997 revealed that individuals with the cognitive ability of self-efficacy may experience fewer negative emotions in emergency situations and, as a result, may feel able to tackle these situations with composure [37]. On the other hand, people who have self-doubts suffer from negative emotions and distress, such as depression. Furthermore, Bandura also stated that individuals with self-efficacy recognize their potential to overcome hurdles and usually focus on problem-solving.

Self-efficacy has been used widely as a moderator in educational and interventional research studies and occupational stress [38,39]. A study in the UK was conducted to assess the depression and stress levels among veterans by using self-efficacy as a moderator. The results revealed that self-efficacy moderated the relationship between combat exposure and posttraumatic stress disorder [9]. Furthermore, the trial of self-efficacy has been reported in healthcare modification, rehabilitation, and disease management in the field of geriatrics [27,40,41].

The main purpose of this study is to examine the effect of COVID-19 fear on the mental health of elderly people and the moderating role of self-efficacy. After a careful review of the literature, we have formulated the following hypotheses:

**Hypothesis** **1:** *Fear of COVID-19 (FOC) has a positive correlation with depression rates amongst the elderly*.

**Hypothesis** **2:** *Exposure to COVID-19 (EC) has a positive correlation with depression rates amongst the elderly*.

**Hypothesis** **3:** *Self-efficacy (SE) has a negative correlation with depression rates amongst the elderly*.

**Hypothesis** **4a:** *Self-efficacy will moderate the relationship between fear of COVID-19 and depression*.

**Hypothesis** **4b:** *Self-efficacy will moderate the relationship between exposure to COVID-19 and depression*.

## 3. Hypothesized Model

**Figure 1:**
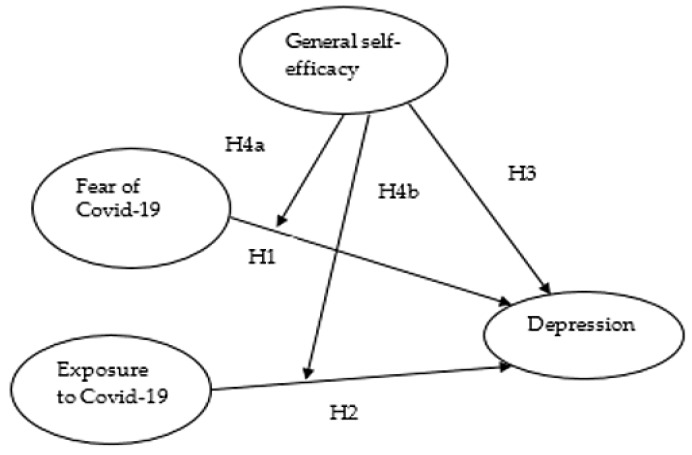
Proposed model of study.

The major objective of this article is to examine the relationship between fear of and exposure to COVID-19 pandemic by checking the influence of self-efficacy as a moderator. According to the evidence from resilience theory and the previous literature review, it is hypothesized that fear and exposure to COVID-19 and self-efficacy are associated positively and negatively with depression, respectively (Hypotheses 1–3, respectively). Furthermore, it is supposed that the association between fear of and exposure to COVID-19 and depression would be less evident among those with high self-efficacy compared to those with low self-efficacy (Hypothesis 4a and 4b, respectively). The hypothesized model of this study has been formulated and shown in Figure 1.

## 4. Methods of the Study

### 4.1. Sample and Data Collection

The first step was the selection of the research participants. Respondents for this research include elderly people aged 50 and above. The time slot of data collection for this study was May 2020–June 2020. The next step was the distribution of the questionnaires. A total of 450 questionnaires were distributed to the target population in Rawalpindi, a metropolitan city in Pakistan. We first developed the questionnaire in the English language and then translated it into Urdu with the help of bilingual experts to ensure content clarity and quality. With the help of a senior researcher, we identified and distributed envelopes to the elderly people who were willing to participate in the study. All respondents were asked to self-administer their responses as honestly as possible and then returned the filled questionnaire to the responsible person.

The number of questionnaires received was 398, a response rate of 84.4%. Later on, 88 incomplete and invalid questionnaires were discarded [42]. The remaining 310 questionnaires were retained, having a response rate of 68.8% for statistical analysis. Respondents were included in the study on the basis of the following inclusion criteria: (i) accepting of the informed consent, (ii) being 50 years of age and above, (iii) being able to read and write in the Urdu language, (iv) living in Pakistan during the COVID-19 outbreak, and (v) not a dementia patient.

### 4.2. Measures and Instruments

This study has used several measurement scales to design a valid questionnaire. Age, sex, and employment status of the respondents were added as socio-demographic variables in the questionnaire. The participants were asked about the fear of virus by using the seven-item “fear of COVID-19 scale” developed by Ahorsu et al. (2020), with α = 0.82 [28]. The sample questions included were: “I am most afraid of coronavirus”; “it makes me uncomfortable to think about coronavirus”; and “my hands become clammy when I think about coronavirus”. The responses were recorded with five options, from “1 = strongly disagree” to “5 = strongly agree”. In relation to the COVID-19 exposure factor, the exposure of the participants to the virus was assessed through the use of two questions; “you are afraid if you′ve been exposed to someone who has COVID-19” and “you are afraid if someone in your family has COVID-19”. These two questions have been used in a previous study under the “direct Exposure to COVID-19” variable [6]. The depression level of the respondents was assessed through the use of the seven-item hospital anxiety and depression (HAD-S) subscale, amongst which questions included “worrying thoughts go through my mind”. This subscale scale was adopted from the original anxiety and depression scale [43] with 0.86 reliability for the depression subscale Iranian validation [32]. Self-efficacy was measured through the use of a ten-item general perceived self-efficacy scale developed and verified by Luszczynska and Schwarzer in 2005 [36], the sample question is as follows: “thanks to my resourcefulness, I can handle unforeseen situations”. All the variables were arranged on the five-point Likert scale response options from strongly disagree to strongly agree. In addition, several demographic variables can have an effect on mental health during COVID-19, such as gender, age, income, and work status [44]. As such, these individuals’ variables may have confounding effects on the results. Therefore, controlling these demographic variables may be useful as they can affect the model [45]. Consistent with our theoretical background, we have used age, gender, and work status as control variables to manage their perplexing prospective effects on our other variables.

### 4.3. Ethics

The current study was approved by the Medical Ethics committee from the Department of Psychological and Behavioral Sciences, Zhejiang University, (reference number: 20-032). All procedures performed in the study involving human participants were in accordance with the ethical standards of the institutional research committee and with the 1964 Helsinki declaration. Consent was obtained from each respondent.

## 5. Results

Data analysis was performed by using the statistical software SPSS, 25.0 version. Multiple linear regression was applied to measure the direct effects of the independent variables on the dependent variable, whereas the moderation effect of self-efficacy was examined by using the Hayes process (v.3) in SPSS [46].

### 5.1. Demographic Assessment

Initially, the socio-demographic assessment of age, gender, and work status was performed by using the frequency analysis as presented in Table 1. Results show that most of the participants (211: 68.1%) were male. About 43% of respondents were 60–70 years of age, and only 7% were above 80 years. Regarding the work status of the respondents, nearly 58% of people were retired or unemployed at the time of the survey.

### 5.2. Descriptive Statistics

The mean, standard deviation, coefficient correlation, and reliability of all the variables shown in Table 2. Fear of COVID-19 was found to be positively correlated with depression (r = 0.146, *p* < 0.05); COVID-19 exposure was also found to be positively correlated with depression (r = 0.203, *p* < 0.01). However, self-efficacy has a negative correlation (r = −0.150, *p* < 0.10) with depression. The alpha reliabilities were above the cutoff value of 0.70 [47,48].

### 5.3. Regression Analysis and Interpretation

The main hypotheses of the present research were tested by multiple linear regression analysis in SPSS 25. This is an extension of the simple linear regression, which is used to check the effects of more than one independent variable in a model [49]. The mean values of the variables were used to conduct regression analysis, the results of which are shown in Table 3. The results of the analysis reveal that the fear of COVID-19 had a significant positive association with depression with the value of (β = 0.162, *p* < 0.05), therefore fully supporting the alternate Hypothesis 1. Similarly, a positive association was also shown between COVID-19 exposure (β = 0.147, *p* < 0.05) and depression; therefore, alternate Hypotheses 2 has also been accepted. The *p*-value ensured a cutoff at 0.05 [50,51]. Further, self-efficacy has a negatively significant association (β = −0.130, *p* < 0.05) with depression, fully supporting Hypothesis 3. Ultimately, the multiple regression results show that the overall research model is significant with (R^2^ = 0.163, F-statistics = 9.852, *p* < 0.05).

We controlled the demographics related to the participants’ age, gender, and work status in the data analysis for other variables, i.e., fear of COVID-19, exposure to COVID-19, self-efficacy, and depression. Nonetheless, the findings showed that control variables have a significant effect on depression.

### 5.4. Moderation Analysis

In the present study, the Hayes process version 3 was used to assess the moderation effect of self-efficacy on the fear of and exposure to COVID-19. Moderation analysis involves the use of linear multiple regression analysis by regressing random variables Y on X; an additional interaction term is added to the model as a new independent variable as (X*M) [52].

Table 4 shows the moderation analysis (Moderation I and Moderation II) results of the Hayes process with the moderation effect of self-efficacy between the exposure to and fear of COVID-19 and depression. In Hypothesis 4a and 4b, we assumed the moderating role of self-efficacy on the relationship between fear and exposure of coronavirus and depression. The results in Table 4 reveal that interaction 1 (FOC*SE) has a positive and significant effect on depression with *p*-value < 0.001, supporting Hypothesis 4a. The *p*-value is less than 0.05, which is often used as the threshold [53,54]. In addition, the interaction term 2 (EC*SE) has an insignificant association with a *p*-value of more than 0.05. Hence, these results do not support Hypothesis 4b. Hence, the outcomes of the Hayes process moderation analysis revealed that self-efficacy moderates the effect on fear of COVID-19 and depression, but it has no significant moderating effect on the exposure to COVID-19 and depression. 

#### Line Graph Interpretation

**Figure 2:**
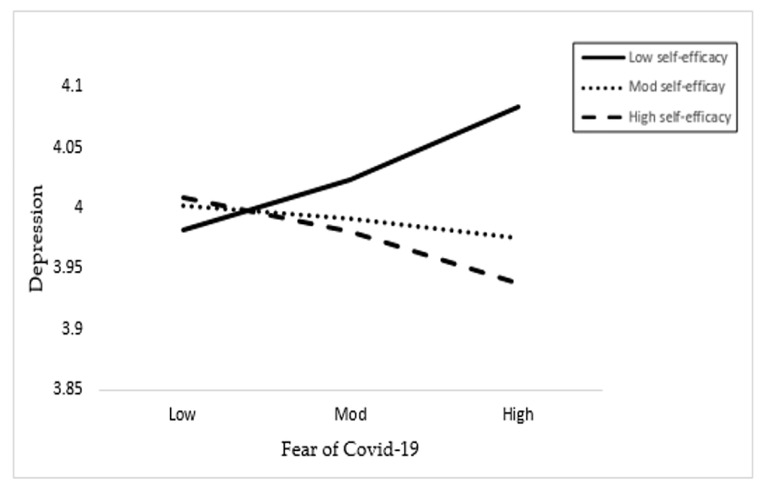
Interactive effect of the fear of COVID-19 and self-efficacy on depression.

**Figure 3:**
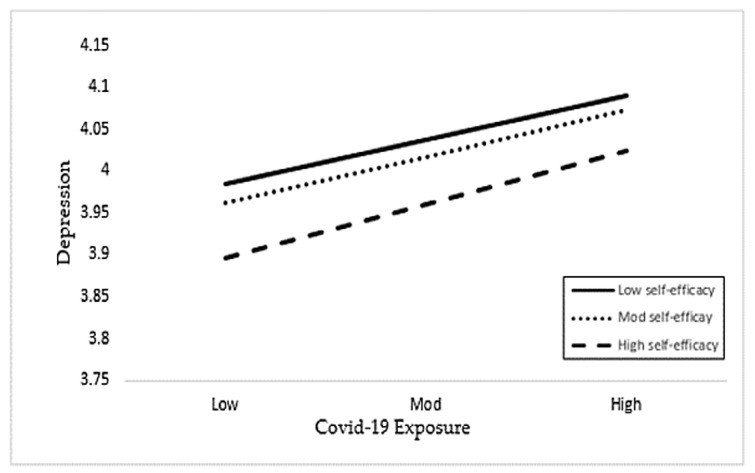
Interactive effect of the COVID-19 exposure and self-efficacy on depression.

**Figure 4:**
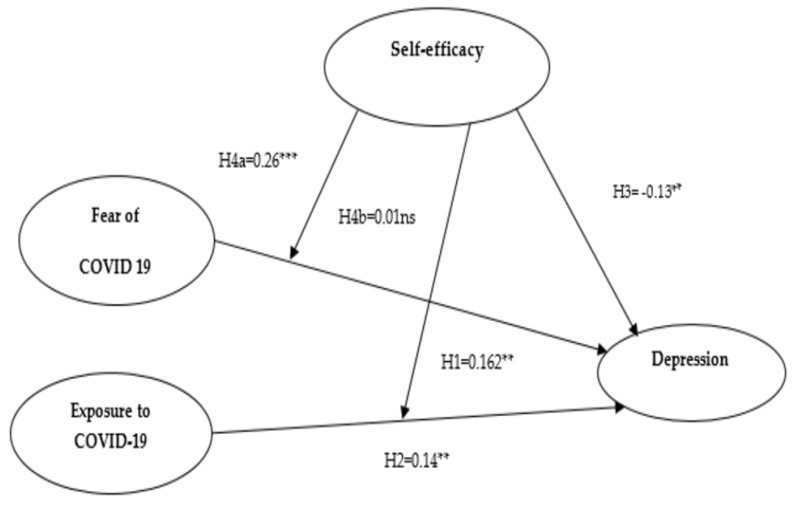
Hypothesized results for the study model (** *p* < 0.01; *** *p* < 0.001; ns = non-significant).

We have plotted the interaction terms in a line graph by showing the three lines as low, moderate, and high values of self-efficacy in relation to fear of and exposure to COVID-19. Figure 2 shows the interaction effect of the fear of COVID-19 and self-efficacy on depression. Figure 3 depicts the moderating effect of COVID-19 exposure and self-efficacy on depression. Figure 4 shows the results of the hypotheses testing.

## 6. Discussion

The main objective of this study was to examine the effect of COVID-19 on the mental health of elderly people with the inclusion of self-efficacy as a moderator. Measuring the effects of both the fear of and exposure to COVID-19 may contribute to the investigation of the mental health of the elderly population during the COVID-19 pandemic. Being a protective component of resilience theory, self-efficacy as a resilient moderating factor helps to identify elderly people’s resilient power against adverse situations.

In relation to our Hypotheses 1 and 2, a positive association was found between depression and both fear and exposure to COVID-19. The positive association between these variables suggests that elderly people were more frequently found to be depressed when they face stressful events, i.e., COVID-19-related fear and exposure in Pakistan. The findings of this study are consistent with previous studies, which identify the presence of fear of COVID-19 as a predictor of depression and anxiety [5], indicating an association between depression and close contact with suspected or confirmed cases [14] and worries surrounding COVID-19 [10]. Furthermore, self-efficacy was also found to be a significant predictor of depression during the pandemic suggesting that self-efficacy ability exerts a strong influence on the depression levels of the elderly population. This finding confirms Bandura’s unifying theory of behavioral change [37], which suggests that people with the self-efficacy abilities may experience fewer negative emotions in emergency situations and are able to tackle these situations with composure.

However, as per our hypothesis, self-efficacy has a significant moderating effect on depression with fear of COVID-19. This moderation role of self-efficacy amongst older people along with a fear of COVID-19 provides additional support for its importance as a resilient force associated with stressors towards better consequences by supporting the resilience theory. This result is in line with the protective factor model of the resilience theory [23], which indicates that the influence of risk factors (e.g., fear of COVID-19) on consequences (e.g., depression) could be buffered by protective factors (e.g., self-efficacy). A Chinese study used self-control as a moderator between the severity of COVID-19 and mental health issues, also reports similar findings that people with less self-control are more likely to suffer mental health issues due to COVID-19 [55].

Our next hypothesis, which states that the association between exposure to COVID-19 and depression would be moderated by self-efficacy and thus elderly people with exposure to COVID-19 and having a strong self-efficacy belief were less likely to suffer from depression, was not supported. Results from the moderation analysis suggests that exposure to COVID-19 may be a more serious threat for elderly people, and their self-efficacy ability does not support them as a resilient factor when they are exposed to COVID-19. A study conducted in the USA supports our finding, where individuals with exposure to COVID-19 were found to be more depressed and concerned [8]. These results seem to contradictory with the findings reported by [56], who revealed that people who are more vulnerable to COVID-19 did not show higher depression levels. Our findings are inconsistent with the conclusions of Wu (2009)—that show that protective measures are often relaxed with family and friends. Nonetheless, fear increases after contact has been made with a COVID-19 patient, with increased feelings of risk, similar to the risk of contracting the virus, also increasing psychosomatic vulnerability [57]. This may be due to the increased fear of transferring the virus to family members after experiencing exposure to the virus.

## 7. Conclusions

To conclude, the findings of this research study confirm the association between COVID-19-related fear, exposure, and the self-efficacy of elderly people with depression during the pandemic in Pakistan. In addition, with the significant association between pandemic-related stressors and depression, the perceived self-efficacy among the elderly population appears to be an influential moderator. Higher self-efficacy ability to control COVID-19-related fear is associated with less depression. Thus, having strong self-efficacy beliefs among elderly people is important to safeguard them against depression. The cognitive abilities of elderly people, such as having a strong belief in oneself as capable of facing stressful events adaptively, seem to affect the relationship between stressors and consequences of COVID-19. Our findings propose that constructing a sense of self-efficacy among the elderly population, through training experiences, may be crucial in ensuring preparedness for high contesting environments and adjustments after such pandemic experiences.

### Limitation of the Study and Future Direction

The nature of our study is cross-sectional and thus does not clearly allow the exploration of the effects of the stressors (e.g., fear of COVID-19 and exposure to COVID-19) on the mental health consequences (i.e., depression) during the pandemic.

The sample design of our study might not be representative of the general population in Pakistan. For instance, we have targeted respondents who are above 50 years of age, with good knowledge of the Urdu language, and without gender distribution. We used a cross-sectional research design, and it is inappropriate to draw causal conclusions. Nevertheless, previous studies provide support for the use of self-designed questions about the exposure to the virus (e.g., [8]), however, it is important to know that the exposure to COVID-19 scale could not be validated previously. Thus, the use of two subjective questions to measure exposure to the virus in the context of friends and family could be a limitation of the study. Future studies may include other variables specific to the older population, such as “has the respondent travelled to highly affected areas or epicentres of COVID-19”, “the effects of media reporting about COVID-19 on depression level”, and “the fear of exclusion due to less social support from family and friends during the pandemic”. Furthermore, the incorporation of more mental health-related variables, e.g., anxiety, trauma, posttraumatic stress disorder, and psychological distress, in relation to the resilience variables may provide insightful empirical and theoretical underpinnings related to the context of pandemics.

Regardless of the study limitations, it cannot be denied that the results of this study gather information regarding the uncertain situation of COVID-19 from the most vulnerable sample of the elderly population, providing vital knowledge about the effects of stressors (i.e., fear of COVID-19, COVID-19 exposure) on the mental wellbeing of older people. In order to respond to pandemic situations, policies should be designed to address not only physical health-related issues but also mental wellbeing. Although having strong self-efficacy ability leads to positive outcomes, fear and exposure to COVID-19 may deteriorate mental health. Measures that obstruct self-efficacy can intensify or worsen consequences, and the level of depressive symptoms may increase. Thus, policymakers may consider self-efficacy as a means of strengthening cognitive abilities, and the importance of resilient factors should be added to the agenda of pandemic prevention strategies.

## Figures and Tables

**Table 1:** Demographic assessment (N = 310).

	Frequency	Percent
**Gender**		
Male	211	68.1
Female	99	31.9
**Age**		
50–60	39	12.6
60–70	135	43.5
70–80	113	36.5
Above 80	23	7.4
**Work status**		
Employed	133	42.9
Retired/unemployed	177	57.1

**Table 2:** Descriptive statistics, reliability, and correlation.

Variable	Mean	Std. D	1	2	3	4
Fear of COVID-19	4.128	0.557	**0.90**			
Depression	3.973	0.665	0.146 *	**0.85**		
Self-efficacy	1.502	0.829	−0.241 **	−0.150 **	**0.94**	
COVID-19 exposure	3.920	1.042	0.203 **	0.129 *	−0.260 **	**0.95**

*, ** Correlation is significant at the 0.01 and 0.05 levels (two-tailed); bold values are Cronbach’s alpha.

**Table 3:** Multiple regression.

Variable	Coefficient	T-Statistic	*p*-Value	Lower Bound CI 95%	Upper Bound CI 95%
Constant	3.321	11.268	0.000	2.706	3.808
Fear of COVID-19	0.162 **	2.582	0.004	0.060	0.311
Self-efficacy	−0.130 **	−2.685	0.008	−0.205	−0.035
Exposure to COVID-19	0.147 **	2.298	0.003	0.050	0.244
Age	−0.105 **	−1.712	0.008	−0.188	0.013
Gender	0.185 ***	3.315	0.001	0.107	0.420
Work status	−0.214 **	−3.576	0.004	−0.445	−0.129
R-square	0.163				
R adjusted	0.147				
F-statistic	9.852 (0.000)				

Dependent variable; depression, ** *p* < 0.01, *** *p* < 0.001.

**Table 4:** Moderation effect.

Moderation Assessment	Coeff.	Std. Error	T-Statistic	*p*-Value	Lower Bound CI 95%	Upper Bound CI 95%	R^2^Δ
Moderation I (FOC*SE)	0.268	0.046	5.743	0.000	0.176	0.359	0.093
Moderation II (EC*SE)	0.012	0.032	0.386	0.699	−0.056	0.075	0.000

Note: SE = standard error; LLCI = lower limit confidence interval; ULCI = upper limit confidence interval. FOC = fear of COVID-19; SE = self-efficacy; EC = exposure to COVID-19.

## Data Availability

The data of this study will be available from the corresponding author (F.M.) upon request.
